# Continuous Production of Prions after Infectious Particles Are Eliminated: Implications for Alzheimer’s Disease

**DOI:** 10.1371/journal.pone.0035471

**Published:** 2012-04-11

**Authors:** Kohtaro Miyazawa, Terry Kipkorir, Sarah Tittman, Laura Manuelidis

**Affiliations:** Section of Neuropathology, Department of Surgery, Yale University Medical School, New Haven, Connecticut, United States of America; Institut National de la Santé et de la Recherche Médicale, France

## Abstract

Rat septal cells, induced to enter a terminal differentiation-like state by temperature shift, produce prion protein (PrP) levels 7x higher than their proliferative counterparts. Host PrP accumulates on the plasma membrane, newly elaborated nanotubes, and cell-to-cell junctions, important conduits for viral spread. To find if elevated PrP increased susceptibility to FU-CJD infection, we determined agent titers under both proliferating and arresting conditions. A short 5 day arrest and a prolonged 140 day arrest increased infectivity by 5x and 122x (>2 logs) respectively as compared to proliferating cells. Total PrP rapidly increased 7x and was even more elevated in proliferating cells that escaped chronic arrest conditions. Amyloid generating PrP (PrP-res), the “infectious prion” form, present at ∼100,000 copies per infectious particle, also increased proportionately by 140 days. However, when these highly infectious cells were switched back to proliferative conditions for 60 days, abundant PrP-res continued to be generated even though 4 logs of titer was lost. An identical 4 log loss was found with maximal PrP and PrP-res production in parallel cells under arresting conditions. While host PrP is essential for TSE agent spread and replication, excessive production of all forms of PrP can be inappropriately perpetuated by living cells, even after the initiating infectious agent is eliminated. Host PrP changes can start as a protective innate immune response that ultimately escapes control. A subset of other neurodegenerative and amyloid diseases, including non-transmissible AD, may be initiated by environmental infectious agents that are no longer present.

## Introduction

It is often stated that the normal host prion protein (PrP) converts itself into an infectious, protease resistant form (PrP-res) that causes diverse transmissible encephalopathies (TSEs). TSEs include human Creutzfeldt-Jakob Disease (CJD) and kuru, sheep scrapie, and epidemic Bovine Spongiform Encephalopathy (BSE). The recent outbreak of a virulent strain of epidemic BSE has now been largely eradicated with the removal of infected meat and livestock. While the infectious protein or prion concept has garnered a large following, *i*) geographically specific environmental origins of different TSEs [1], [2], [3], *ii*) the classic viral-like spread of these agents through the lymphoreticular system, white blood cells and dendritic cells to the brain [4], [5], *iii*) early innate immune responses of the host that signify host recognition of a foreign infectious agent [6], *iv*) abundant agent replication before PrP-res becomes detectable [7], [8], and *v*) the lack of any consistent strain-specific forms of “infectious” PrP [1], [9] all pose fundamental problems for the prion hypothesis. For simplicity, we here consider prions as their originally defined PrP^sc^ form (identical to proteinase K resistant PrP-res) [10].

A variety of independent experimental approaches have been used to determine the quantitative relationship of PrP-res to the number (titer) of infectious particles. While PrP-res is a valuable diagnostic marker for TSE disease, analytic experiments have repeatedly shown that PrP and PrP-res quantities are poor predictors of infectious titer. For example, infectious ∼25nm virus-like particles reproducibly separate from the majority of PrP and PrP-res during centrifugation (reviewed in [11]), and test-tube PrP misfolding-conversion “PMCA” assays typically show enormous amounts of *de novo* PrP-res can be generated with low or absent infectivity (e.g., [12], [13]). PrP-res itself appears to be insufficient for infection and most PMCA reactions need to be repeatedly primed with complex brain homogenates. Cell free replication does not rule out a viral structure because plant and even mammalian viruses such as poliovirus [14] can make substantial infectious virus in cell free systems; presumably even more virus would be produced if the needed cell free components for viral synthesis or assembly were similarly replenished. The reported generation of infectivity from recombinant PrP alone [15] has not been reproduced, nor has anyone independently repeated the high infectivity from recombinant PrP and lipids without the borrowed and pooled infectious “seed” [16]. Furthermore, recent critical experiments have demonstrated inadvertent contamination underlying “spontaneous conversion” into an infectious form [17].

To account for the discrepancy between PrP-res and infectious titer, it is now postulated that a minor, and still uncharacterized protease-sensitive form of PrP, is the real infectious entity [18], [19]. To test this suggestion, we analyzed brain and cell homogenates before and after proteolytic digestion for all detectable forms of PrP as well as for infectivity. Remarkably, virtually complete digestion of all forms of PrP in high titer FU-CJD infected brain homogenates did not reduce the infectious titer [20]. While it can be argued that only a minute number of invisible prion molecules constitute the infectious entity, it is more likely that PrP is not the infectious agent, but instead, the essential host receptor for a foreign infectious particle that contains a nucleic acid genome. Indeed, circular DNAs with long sequence stretches not in the database have been identified in a variety of infectious preparations [21]. The idea that PrP is a TSE virus receptor readily accounts for the failure of TSE agents to infect PrP knockout mice. Although the normal function(s) of PrP is not entirely clear, cell and developmental biology experiments also show that PrP normally functions as a membrane receptor, one that additionally may act as a crucial facilitator for transmission of infectious TSE particles from cell-to-cell [22]. A dramatic rise in PrP occurs when embryonic rat neural cells are induced to differentiate by proliferative arrest. Concomitantly, these arrested cells develop extensive PrP-rich cell-to-cell junctions, in addition to a network of connecting nanotubes, structures known to transport and spread viruses throughout a cell population (reviewed in [22]). On the other hand, PrP can also be part of an innate immune defense mechanism. A rapid PrP-res rise in response to infection by the kuru agent in the GT1 neural cell line appears to prohibit accumulation of infectious particles. In contrast, very high FU-CJD agent titers are found in GT1 cells when PrP-res responses are delayed, suggesting PrP can help to retard or diminish infection [23]. In accord with this, PrP has antiviral properties and reduces HIV expression [24]. PrP also displays wider antimicrobial activities [25] that are most consistent with innate immune anti-infective functions.

To further test the TSE-agent-PrP dependent receptor concept, and clarify if PrP has a role in host defense, we evaluated the temporal relationship between agent titers and PrP in both proliferating low PrP, and arrested high PrP rat neural progenitor septal (SEP) cells. SEP cells carry the temperature sensitive SV-40 T antigen that provides for proliferative arrest at the physiological temperature of 37.5°C in 2% serum; proliferative SEP cells are kept at 33°C in 10% serum. For infection, we used rat brain homogenates carrying the Japanese human FU-CJD agent, a strain that is more virulent than the classic sporadic CJD (sCJD) agent found in the USA and Europe [2], [23]. By the 3^rd^ serial rat passage (p3), the more virulent FU-CJD agent achieved an incubation time in rats that was ∼100 days shorter than in sCJD rats ([8] and LM unpublished data). Interestingly, as found with other TSE agents transmitted to heterologous species, the rat-propagated FU-CJD agent maintained its ability to infect murine GT1 cells with the same characteristics as the FU-CJD propagated in mice. Thus GT1 cell assays yielded reliable titrations of FU-CJD particles in SEP cells.

We here show that 1) a short 5 day induction of high PrP by SEP cell arrest leads to a small 5x increase in infectivity over parallel proliferating low PrP SEP cells; 2) cells chronically maintained under arresting conditions for >100 days show a dramatic >2 log increase in titer compared to proliferating cells, and 3) infected chronically arrested cells, when returned to a proliferative state, maintain very high levels of both PrP and PrP-res even though almost all infectious particles have been eliminated. The large amounts of residual PrP-res that continue to be produced in such “self-cured” cells provides a living cell parallel to the generation of non-infectious PrP-res in PMCA test-tube assays. Living cells therefore can independently perpetuate a cascade of PrP amyloid without infection, and with no requirement for inflammatory cells. Although PrP aggregation and amyloid formation initially may be part of a protective, innate cell response to infectious particle binding, PrP misfolding appears to develop progressively, and then continues to be formed as an inappropriate end-stage reactive cascade, much like that seen in auto-immunity. These findings raise an important new perspective on the origin and development of more common neurodegenerative amyloid diseases, including non-transmissible Alzheimer’s Disease (AD) [26]. A subset of AD brains may have amyloid and other protein aggregates caused or initiated by hidden and/or previously cured viral infections [27]. Indeed, neurodegenerative amyloids, including PrP-res, are typically late-stage pathologic products that can be triggered by one or more environmental factors.

## Materials and Methods

### Infectious Material and Established Cell Lines

All animal procedures were carried out in strict accordance with the recommendations in the Guide for the Care and Use of Laboratory Animals of the National Institutes of Health (NIH) and Yale Animal Care approved protocols and are in compliance with all ethical standards. Yale is an Association for Assessment of Laboratory Animal Care (AALAC)-accredited facility and the protocol used was approved by the Yale Institutional Animal Care and Use Committee (IACUC protocol #2011–10020). All procedures were done under approved anesthesia under best practice at the time when collected here and as published [8], [9], and all efforts were made to minimize distress. The established cell lines and sources have previously been reported for all lines used, including embryonic rat septal (SEP) cells originally started by B. Wainer [28] and the murine hypothalamic GT1 cell line of Nishida [2], [9].

### Conditions for Infection, Proliferation and Arrest

**Figure 1:**
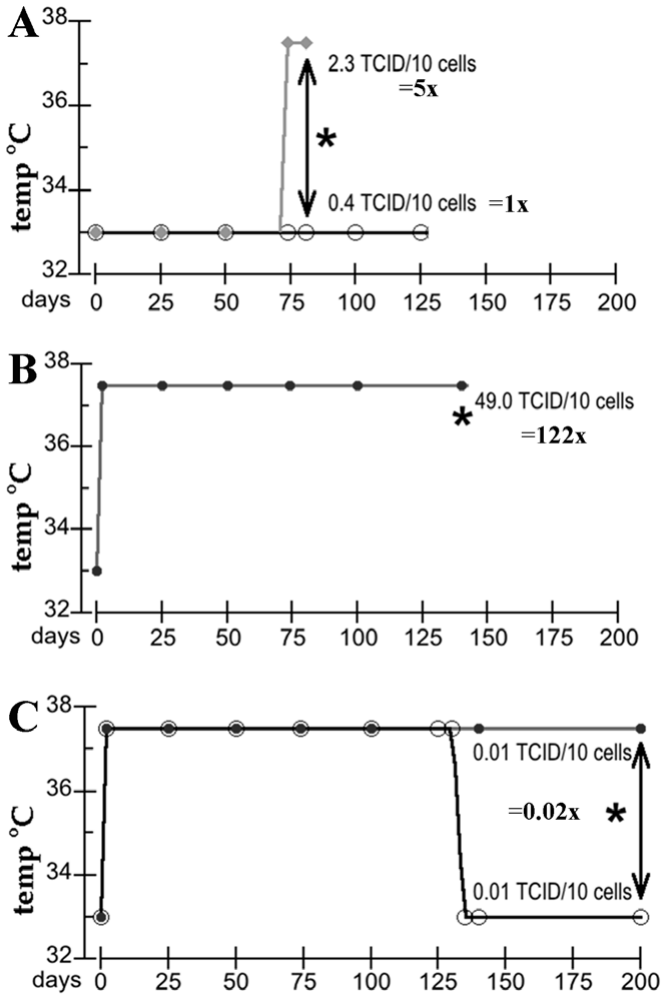
Outline of the three temperature shift experiments with days post-infection (p.i.) sample points analyzed for PrP and PrP-res. The * indicates tissue culture infectious dose (TCID) determined at the indicated time points. **A**) infected proliferating SEP cells at 33°C-10% serum with parallel cells arrested at 37.5°C-2% serum for 5 days; the latter cells contained 5x more infectious particles. **B**) cells infected at 37.5°C-2% serum (p0) and maintained under arresting conditions for a total of 140 days. Cells at 140 days contained >2 logs more infectivity than control proliferating cells in A. **C**) comparison of parallel aliquots of high titer cells returned to proliferative conditions, or maintained under arresting conditions until 200 days. TCID determinations were done for both sets at 200 days, and both showed an ∼4 logs (5,000 fold) reduction in infectious particles per cell as compared to 140 day precursor cells. For examples of the TCID assay see Fig. 4.

Low passage rat neural progenitor SEP cells (subclone e422, a gift of B. Wainer) as published [28], were maintained and assayed as previously described under verified proliferative and stationary conditions [22]. Briefly, proliferating cells were maintained at 33°C with 10% fetal calf serum in DMEM, whereas stationary arrest was induced by elevating the temperature to 37.5°C and reducing serum to 2%. Proliferating cells were split 1∶4 every 4 days. Stationary cells were 90% arrested by day 2, as previously shown by uptake of BrdU with a corresponding decrease in SV40 T antigen; concomitantly, there was a rapid 5–10 fold increase in prion protein (PrP) detected on Western blots, with obvious PrP accumulations at the cell surface [22]. Stationary cells were initially refed in situ, and then split at 1∶2 every 7 days up to p11 (day 81). After this, cells under stationary conditions could be split at 1∶4 every 4 days because cells not completely arrested had a growth advantage that allowed them to predominate. As graphically detailed, a short 5 day arrest of infected proliferating cells (Fig. 1A) was compared to long term infection under continuous arresting conditions for 140 days (Fig. 1B). Chronically “arrested” cells were also compared for 60 additional days to parallel cells returned to proliferative conditions at 140 days (Fig. 1C).

Infection of immortalized SEP cells were performed as previously for other cell lines here [9] with minor modifications. Briefly, before infection, e5 cells were plated in each well of a 6-well chamber plate. At day 3, cells were exposed to infected FU-CJD rat brain (10 µl of a 10% brain homogenate in 2 ml medium). Two days after application of infectious material, cells were washed to completely remove residual brain (including PrP and PrP-res) as previously demonstrated [29], and fresh medium was added for an additional 3 days. For proliferating cells, the first post-infection (p.i.) passage (p1) split was at 1∶4. Three different frozen FU-CJD infected rat brain samples (from rat serial passages 3 and 4) all gave positive *de novo* PrP-res signals after SEP cell infection. Control SEP cells analyzed before infection at day 0 showed no PrP-res. For infection of arrested cells, the same basic procedure was used, but the first day after seeding at 33°C-10% serum, cells were transferred to 37.5°C-2% serum for 2 days to induce arrest with high PrP during infection. These stationary cells were then exposed to FU-CJD rat brain homogenate for 4 days before being washed, refed in situ, and then split at 1∶2 every 7 days until p11 as detailed above.

### Protein and Infectivity Assays

Standard Western blotting with quantitative chemiluminescent detection of signals was done using the primary commercial antibodies for PrP, tubulin and ß-actin as previously detailed [9], [22]. The tissue culture infectious dose in each SEP cell homogenate was assayed by exposing duplicate wells seeded with murine GT1 indicator cells to a series of SEP homogenate dilutions. Dilutions of FU-CJD infected rat SEP cells showed the same strain-specific progressive induction of PrP-res as FU-CJD infected mouse brain and murine GT1 hypothalamic cells [23]. Serial dilution assays also showed that FU-CJD infected SEP rat cells were capable of producing very high titers of infectious particles. As many as 5 TCID per cell could be produced under arresting conditions, (i.e., 5e9 TCID per gram brain with e9 cells). This is comparable to the high titer of FU-CJD GT1 murine cells (3 TCID per cell). The TCID in rat SEP cells was accordingly calculated using the previously defined standard FU-CJD agent-specific curves [23]. For example, at p7 the TCID  = 71.89*e^ (0^ ^23436*X)^, and at p5 TCID = 10.781*e ^(0^ ^25224*X)^; the slopes of both lines are the same at different passages but the infectivity is less when the same %PrP-res requires more passages. Average %PrP-res of duplicates were used for all TCID determinations, and corresponded to plots shown previously for each FU-CJD passage [23]. For reference, tissue culture infectious doses (TCID) are ∼2–3 fold less than determined in animals by LD_50_, probably because mice are followed for a much longer time, up to 500 days p.i. for end-points.

## Results

If host PrP is a required receptor for TSE agents, then increasing the level of PrP should increase both susceptibility to infection and the infectious titer. In Tga20 mice with 8x the levels of PrP, the incubation time of CJD and scrapie agents is reduced as compared to wt mice with 1x PrP [1], [3], [9], [30]. This shows PrP enhances susceptibility to TSE infectious agents. On the other hand, the final brain titer in Tga20 mice is not higher than in wt mice [30]. Rat SEP cell cultures that will produce high PrP levels under arresting conditions (37.5°C-2% serum) offered another opportunity to explore the relationship of titer to PrP and PrP-res in a highly controlled biological system. Uninfected SEP cells rapidly transition into a stationary non-dividing state that resembles terminal neural differentiation, and they concomitantly express ∼7x levels of PrP. When uninfected control SEP cells are maintained long term at 37.5°C-2% serum they continue to produce 5–8x levels of PrP, and when switched to standard proliferative conditions (33°C-10% serum) they immediately revert to 1x PrP levels [22].

Fig. 1 shows the basic design of experiments where infectivity and both PrP and PrP-res were analyzed. In the first strategy (Fig. 1A) proliferating SEP cells were exposed to FU-CJD brain homogenates, grown and split for 15 passages (day 74 p.i.) and then switched to the arresting conditions of 37.5°C-2% serum for 5 days. As indicated, proliferating counterparts (33°C-10% serum) were compared in parallel at the same passage. The extended maintenance of proliferating infected SEP cells amounted to a >1 in a trillion dilution of p1 cells under standard 1∶4 dilutions at each passage. Tissue culture infectious dose assays (TCID) revealed a stable and continuous baseline of productive infection in proliferating SEP cells (0.43 TCID per 10 cells). Acutely arrested SEP cells demonstrated a 5x increase in FU-CJD agent as compared to their proliferating counterparts. As indicated in Fig. 1A, these stationary cells contained 2.3 TCID per 10 cells. This small increase was reproduced with several different FU-CJD rat brain homogenates and could be due to increased accumulation of infectious particles in non-dividing cells. Since the effective doubling time of the FU-CJD agent in monotypic cells is 1 day [23], one would predict an 8x increase in titer during the 3 days after SEP cells stopped dividing, in reasonable accord with the 5x increase found. Arrested cells were healthy and comparable to proliferating infected and uninfected controls.

To find if arrested SEP cells with high PrP are more susceptible to infection than proliferating cells, and if higher levels of infection can be propagated under chronic arrest conditions, we used the strategy shown in Fig. 1B. Previous control experiments showed that uninfected SEP cells grown under arresting conditions maintained high PrP levels long term, even after the small population of dividing cells began to dominate. FU-CJD infected cells arrested for a prolonged time period also revealed that selected cells progressively escaped arrest. Infected arrested cells could be split at only 1∶2 for 11 passages, but after this could be split at 1∶4 despite continued maintenance under arresting conditions. This is not surprising because SEP cell arrest is not absolute, and 5–10% show nuclear DNA synthesis with a 2 day arrest [22]. SEP cells were maintained at 37.5°C-2% serum for a total of 140 days (p19), before collecting cells for TCID determinations. By 140 days p.i. the total original infected cells that remained accounted for only 1 in a population of a million. These cells that escaped arrest showed no toxic or morphological changes as compared to controls. However, they contained 49 TCID per 10 cells, a huge 122x increase in infectivity over proliferating cells (Fig. 1A). Additionally, cells under long-term arresting conditions showed a significant increase in infectivity compared to 5-day arrested cells (21.3x). Thus the long term increase of infectious particles was clearly not due to a simple accumulation of particles in non-dividing SEP cells.

It was relevant to find if infectivity is reduced after reversal of long-term arresting conditions. Fig. 1C shows this third strategy. Because uninfected SEP cells with high PrP rapidly reverted to a low PrP state when switched to proliferative conditions, we expected the highly infectious SEP cells here would show the same immediate reversion to a low PrP state. A concomitant reduction in infectious particles to baseline levels would also be expected. When long-term arrested cells were returned to proliferative conditions at day 135 (p18), and then maintained until day 200 p.i. (p28), they displayed markedly reduced infectivity, with only 1 TCID per 1,000 cells, considerably less (1/40^th^) than the infectivity of infected basal proliferating cells (see Fig. 1A). Unexpectedly, arrested SEP cells maintained in parallel for 200 days (to p28) at 37°C-2% serum also revealed an identical negligible number of infectious particles even though they appeared indistinguishable from high titer cells. Indeed, these arrested cells maintained only 1 in 4,900 of the particles of their 140 day precursors (compare Fig. 1B). Presumably, according to the prion hypothesis, the released proliferating SEP cells, as well as those maintained under arresting conditions, should concomitantly show negligible PrP and/or PrP-res.

**Figure 2:**
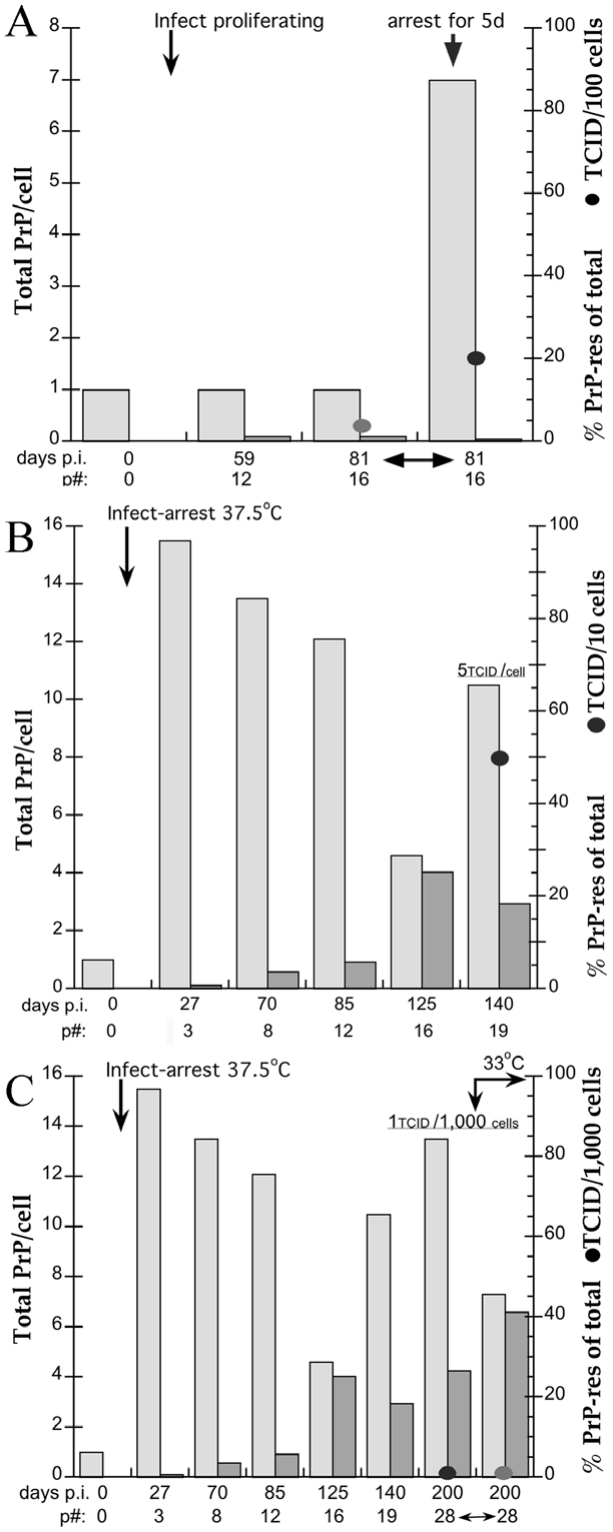
Total PrP (light gray bars) and %PrP-res of the total PrP (dark gray bars) at progressive times corresponding to outlined experiments A, B, and C in Fig. 1. Note the TCID scale change in each graph. Prior to infection (p0), proliferating SEP cells have a baseline of 1x total PrP and no detectable PrP-res. **A**) Post-infection with FU-CJD, proliferating cells showed continuous production of low amounts of PrP-res (1.2% of total PrP) up to 125 days; these proliferating infectious cells were compared at 81 days with parallel SEP cells that were switched in parallel for 5 days to arresting conditions. PrP-res was 0.6%, and total PrP had increased 7-fold in 5 days; previous 5 day arrest of uninfected SEP cells showed the same 7-fold rise in total PrP, but no PrP-res [22]. **B**) With long-term arresting conditions, total PrP remains very elevated (>10x of proliferating cells); the single 125 day sample with a lower total PrP contained a relatively high %PrP-res, consistent with breakdown by cellular proteases; these PrP changes were not a consequence of long term arrest because the subsequent 140 day arrested cells again contained markedly elevated PrP. Note the slowly progressive increase in %PrP-res and the corresponding high 5 TCID/cell after 140 days of arrest. **C**). With continued arrest to 200 days, total PrP was again very elevated (13.3x) and PrP-res was even higher than at 140 days. Unexpectedly these cells contained only 1 TCID/1,000 cells, i.e., almost 4 logs of infectious particles were eliminated. FU-CJD infected cells switched for 60 days to proliferative conditions also continued to produce high PrP, unlike previously documented uninfected controls [22], and also continued to convert PrP into very high levels of PrP-res (40% of total PrP). TCID are shown by filled circles (note change in scale between A,B and C).

**Figure 3:**
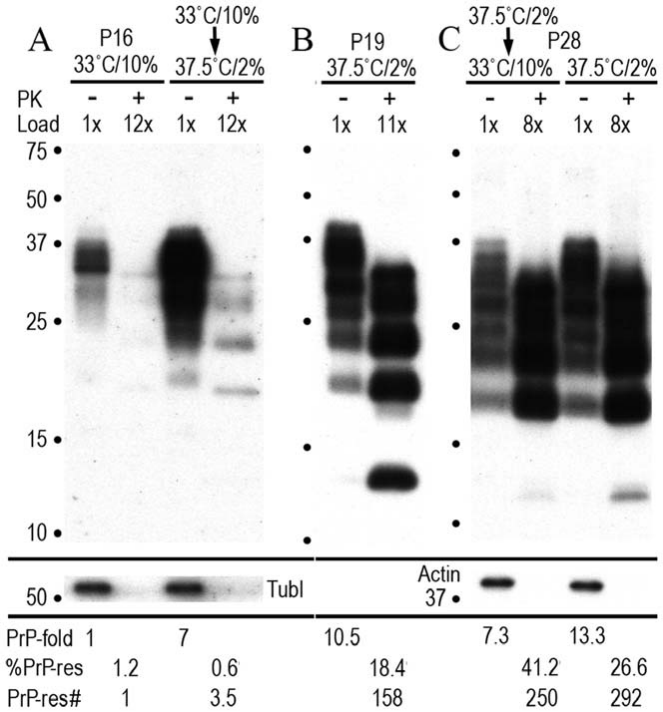
Representative Western blots of PrP and PrP-res at the time of infectivity assay in outlined experiments A, B, and C. Note the unchanging 13kd band of PrP-res that is a unique characteristic of Asiatic FU-CJD agent strain in all blots, including the 200 day (p28) samples. **A**) At p16, FU-CJD infected proliferating cells at 33°C-10% serum have baseline (1x) low amounts of total PrP. Abnormal PrP-res was visible only on longer exposures and accounted for 1.2% of total PrP in these proliferating cells. With a short 5 day arrest, the total PrP rose 7-fold, with 0.6% PrP-res as indicated under each lane. The relative increase in PrP-res molecules (PrP-res #) is also shown in comparison to proliferating cells. Tubulin was used to normalize loads, and as shown, was equivalent in both undigested samples. The tubulin signal was abolished by PK treatment for PrP-res. **B**) shows high PrP and PrP-res after continuous long-term arresting conditions (p19 in 37.5°C-2% serum) and is also indicated quantitatively under the lanes. **C**) Long-term arrested cells switched to proliferative conditions, as well as those continued in parallel for 60 days (to p28) in arresting conditions, both display elevated PrP and PrP-res as indicated. The relative number of PrP-res molecules was even higher than in the precursor 140 day cells with 4 logs more infectivity. Molecular size markers indicated by dots.

Fig. 2 shows the total PrP and %PrP-res during the three temperature-serum shift experiments outlined above. In Fig. 2A, no PrP-res was detectable in proliferating cells (day 0, p0) prior to infection. After infection, total PrP in the proliferating cells did not change, and only a small amount of PrP-res (1.2% of total PrP) was detectable for up to 81 days p.i. With a short 5 day arrest at 37.5°C-2% serum, infected SEP cells, as previous control uninfected cells [22], rapidly displayed a 7 fold increase in total PrP, 0.6% of which is PrP-res. Western blot primary data for this p16 time point is shown in Fig. 3A with tubulin used to normalize protein loads in each lane. While the %PrP-res did not correlate with the acute 5x increase in TCID at 5 days (solid circles), it did show a reasonable correlation to the relative increase in the number of PrP-res molecules compared to proliferating controls. There was a 7x increase in total PrP and a 3.5 fold relative increase in PrP-res molecules (see Fig. 3A, PrP-res #). This number is biologically equivalent to a 5x increase in infectious particles and represents an increase of ∼100,000 PrP-res molecules per TCID. Note the 13kd band seen in FU-CJD infected SEP cells, an agent specific marker that is induced only by Asiatic CJD isolates propagated in cell cultures [3].

Long-term propagation of FU-CJD SEP cells under arresting conditions documents the continuous maintenance of high PrP levels. Cells were sampled representatively between 27 and 140 days p.i. as shown in the bar graph of Fig. 2B. During this extended arrest, the total PrP in FU-CJD infected SEP cells was, with one exception, 10–16x higher than in parallel infected proliferating cells. Interestingly, uninfected controls during long term arrest showed an average of 6.8x (+0.7 SEM) total PrP whereas FU-CJD infected cells, even with the inclusion of the single low outlier, had a higher average total PrP of 11x (+1.4 SEM). This was statistically significant by student’s t-test, with a mean difference of 4.1x (P = 0.03). It thus appears that chronic TSE infection of neural SEP cells, without the contribution of inflammatory or other cell types, is capable of inducing a distinct PrP response that exceeds that found in standard differentiation arrest.

Unlike test-tube PMCA experiments where PrP rapidly converts to PrP-res, the PrP-res in cells accumulated slowly and progressively. PrP-res rose from 0.7% PrP-res at day 27 (p3) to 18–25% by 125–140 days. This slow accumulation of PrP-res could suggest PrP-res was elaborated as part of a defense mechanism against increasing accumulation of infectious particles. At 140 days, the %PrP-res was 18x greater than in proliferating SEP cells that contained 2 logs less infectivity. However, because the total PrP was 11x greater than in the FU-CJD proliferating cells, there was a 158 fold increase in the relative number of PrP-res molecules. Thus this example does not distinguish PrP-res from infectious titers; the 158x proportional increase in PrP-res molecules fairly reflects the 122x increase in titer. Fig. 3B shows the Western blot of this 140 day (p19) sample with very high infectivity (5 TCID/cell). The more rapid proliferation of arrested SEP cells after p11, equivalent to a SEP cell dilution of ∼100,000, clearly did not inhibit the enormously increased production of infectious particles, nor did it reduce the total PrP elaborated. This titer increase further indicates an enhanced cell-to cell transit of infectious particles that is facilitated by high plasma membrane PrP, and/or a progressive replication of infectious particles boosted by continuous high PrP availability. Note again the induced 13kd PrP band that is agent-specific and has not changed with 140 days of replication in SEP cells.

While an increase in total number of PrP-res molecules showed a reasonable correlation with infectivity in the first two experiments, the third strategy uncovered a profound dissociation. This experiment demonstrated that 1) changes in PrP are part of an accelerating host response to infection that could not be shut off, and 2) markedly abnormal PrP-res accumulations were maintained after virtually all infectious particles were eliminated. Previous independent experiments have shown that all forms of PrP can be destroyed without loss of infectivity [20], and that dendritic-like myeloid microglial cells without detectable PrP-res are highly infectious [31]. However, abundant production of PrP-res in a living cell without significant infectivity has not been reported previously. This is important because PrP-res is often considered synonymous with infectious titers. The following data underscore PrP and PrP-res changes that are part of a pathological host response to infection rather than the causative infectious agent.

Two aliquots of ells maintained under arresting conditions for 135 days (the same as those producing the very high titer of 5 TCID/cell at p19, only 5 days later) were used for further passages. Half of these SEP cells were maintained under arresting conditions for 10 additional passages until day 200 (p28). The other half was switched to proliferative conditions and grown in parallel from p19 to p28 as diagrammed in Fig. 1C. The cell dilution between p19 and p28 was 3 million, and thus the titer of the released proliferating cells should be significantly reduced. However, as noted above, the titer in these cells was even lower (1 TCID/1,000 cells) than in proliferating controls. According to the prion concept, one would also expect the relative number of PrP-res molecules to be reduced to 0.0025x of the baseline proliferating controls. As shown in Fig. 2C (bars under 33°C) the %PrP-res at p28 still remained markedly elevated and accounted for 41% of the total PrP. Moreover, the total PrP remained much higher than seen in either uninfected controls or in infected proliferating cells (compare 2A and 2C). As documented in the corresponding Fig. 3C Western blot, the total PrP was only slightly reduced, and remained 7.3x the normal proliferative levels even 60 days after release in 33°C-10% serum. The number of PrP-res molecules in these cells with negligible infectivity, moreover, was 1.6 fold higher than found in their high titer 140 day predecessors. Additionally, the relative number of PrP-res molecules was also 250 fold greater than the more infectious proliferative cells at p16 (compare Fig. 3A and 3C lanes at 33°C). Notably, there was no evidence of any change in the FU-CJD agent strain from PrP and PrP-res characteristics; The FU-CJD strain, as does another Asiatic isolate (YAM), still induced the strain specific 13kd PrP-res band at 200 days. Clearly, infection with the FU-CJD agent can set off a cascade of PrP changes that once established, can not be readily reversed by either environmental conditions, or even by the extraordinary elimination of ∼4 logs of infectivity.

This concept was further extended by the analysis of cells maintained under arresting conditions from 140 to 200 days. As shown in Fig. 2C cells at 200 days (p28) appeared indistinguishable from p19 cells at 140 days with respect to both total PrP and PrP-res, banding patterns, and morphological and growth characteristics. Total PrP remained elevated at 13.3x, an even higher level than seen in the highly infectious cells at day 140. PrP-res additionally became even more abnormally elevated and accounted for 26.6% of the total PrP. The evidence for this is detailed in the corresponding Western blot lanes of Fig. 3C. With a relative PrP-res number of 250, regardless of the form of PrP, they should have equal or higher levels of infectivity than their 140 day precursors. Yet the titer was reduced by ∼4 logs, an identical loss of infectivity as their parallel counterparts switched to proliferative conditions. This finding cannot be explained by a simple retention of cytoplasmic PrP-res from day 140 because the 9 additional 1∶4 cell splits between 140–200 days should also have diluted PrP-res by 3 million fold to undetectable amounts. Again, these cells continued to produce abnormal PrP-res aggregates completely out of proportion to their negligible infectivity. This continuous production of PrP-res underscores a biological host cell reaction that may have started as a protective response, but has escaped control.

**Figure 4:**
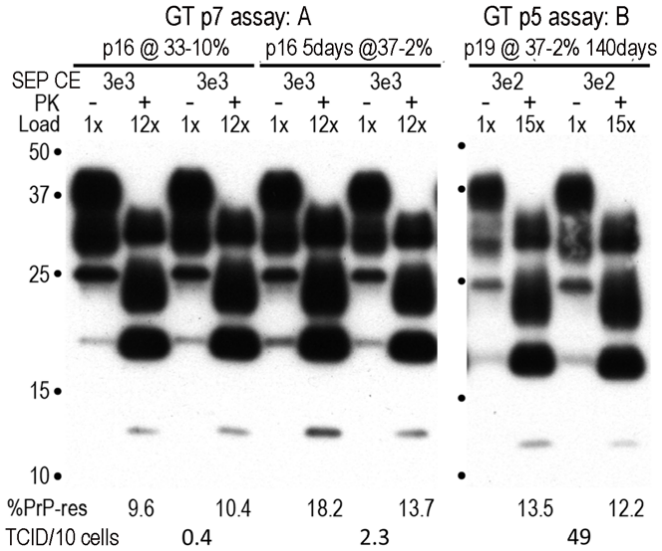
Examples of infectivity assay with relative TCID per cell determined by serial SEP cell dilutions. CE are the number of cell equivalents applied to target GT1 indicator cells, with the %PrP-res and corresponding TCID noted below the sample lanes. In cells from experiment A, an input of 3e3 SEP cells and 7 passages were required to elicit substantial PrP-res production in GT1 indicator cells. The PrP-res in the 5 day arrested cells is higher than in the proliferative cells, with a corresponding 5 fold increase in infectious particles. In contrast to experiment A, 10-fold fewer (3e2) chronically arrested SEP cells (140 days, experiment B) elicited a substantial PrP-res response much earlier, at assay p5. CE dilutions of the rat passaged FU-CJD agent again showed the same FU-CJD specific 13kd band in murine GT1 cells despite passaging in rats. Moreover, the strain-specific replication curve was identical to the FU-CJD agent propagated in mice, i.e., this strain is very stable and showed no detectable changes even though species-specific host responses to this agent are quite distinct in terms of incubation time (120 days in mice versus 222 days in rats) and neuropathology (LM, unpublished data).

Fig. 4 shows representative TCID assays of SEP cell homogenates applied to indicator GT1 cells. In these assays, serial dilutions of FU-CJD SEP cells at known inputs or cell equivalents (CE) were applied to GT1 target cells. The time (passages) required to elicit a PrP-res response to the FU-CJD agent was analyzed. High titer samples elicit a PrP-res response at early passages whereas low titer cells need a greater CE input to induce the same level of PrP-res accumulation in GT1 indicator cells. After a short 5 day arrest (Fig. 4A), it required 7 passages of GT1 cells, and an input of 3e3 CE of infected SEP cells to detect a substantial PrP-res response. These 5 day arrested SEP cells (37–2% lanes) elicited more PrP-res in the indicator GT1 cells than their 33–10% SEP counterparts, equivalent to a small but reproducible 5x increase in titer. Fig. 4B shows that application of 10-fold fewer SEP cells (only 3e2 CE) gave a strong PrP-res response after only 5 GT1 passages, equivalent to 5 TCID per cell. In contrast, 1,000 fold more SEP cells (3e5 CE) collected at 200 days were required to produce a lower PrP-res signal, even after 7 passages (data not shown).

## Discussion

FU-CJD infection of living SEP cells provides insight into innate host cell responses that have been difficult to sort out in animal models with complex and multiple cell type reactive changes. The cell biology of infection and control are not operative and cannot be analyzed in test tube reconstitution experiments such as PMCA. The production of extremely high levels of FU-CJD infectivity in SEP cells with ∼10x normal levels of PrP was unexpected because Tga20 mice with comparably high PrP levels have not shown increased titers compared to wt mice with 1x PrP; this is true for diverse agents including Chandler (RML) scrapie, FU-CJD, and kuru agents in Tga20 mice [3], [23], [30]. On the other hand, both SEP cells and Tga20 mice show high PrP facilitates infection. In the case of Tga20 mice, this is reflected by the more rapid progression to terminal disease, with relatively short incubation times as compared to wt mice. Similarly, the short 5 day SEP cell arrest with elevation of PrP led to an increase of the FU-CJD infectious agent close to the predicted level based on the FU-CJD doubling time. This data is in keeping with PrP as a receptor that binds to and facilitates the entry and reproduction of the TSE infectious particle. In mice, high levels of infectious particles will produce clinical signs of disease, including host astrocytic and microglial responses that can be destructive and lead to death [8], [32]. Even higher levels of agent did not kill the SEP cells here, possibly because they are not subject to destructive cellular elements. SEP cells maintained for 140 days under arresting conditions continued to produce infectious particles and high PrP levels even though they escaped arresting conditions and proliferated at the same rate as their untreated counterparts. Remarkably, titers of these proliferating FU-CJD infected, high PrP SEP cells were ∼2 logs greater than found in RML scrapie brain, i.e., 9.3 versus 7.3 logs per gram (e9 cells). This difference may, at least in part, be due to host responses to the infectious agent before PrP-res is detectable. During the initial phase of *in vivo* infection, non-PrP host responses clearly recognize the infectious agent [6]. Moreover, agent doubling time studies show that it can take as much as 22x longer to accumulate the same number of infectious particles in a mouse brain as compared to cultured monotypic cells [23]. This emphasizes host recognition of and defense against an invading foreign TSE agent.

SEP cells also revealed a proportional and progressive rise in the relative number of PrP-res molecules that accompanied titer increases in 2 of the 3 experiments. The meaning of this PrP-res accumulation is not entirely clear because in absolute terms PrP-res molecules were present at >100,000 per infectious particle. The %PrP-res rose progressively from 0.6% to >20% of total PrP in cells kept for 140 days under arresting conditions. On the other hand, in Tga20 mice the number of PrP-res molecules never exceeded the numbers found in wt mice, i.e., there is a much lower %PrP-res in high PrP Tga20 mice than in wt mice. In contrast to infected Tga20 mice where baseline PrP is not increased, total PrP was significantly higher in infected versus uninfected SEP cells. Continuous production of higher PrP was maintained under prolonged arrest. This indicated that FU-CJD infection itself can induce the host to synthesize additional PrP as part of a protective response. Since increased total PrP was not seen in proliferating cells with low infectivity, relatively high levels of infectivity appear to be required to enhance PrP production, an observation in keeping with a protective role for PrP. A protective role for PrP has also been noted in various types of infection [24], [25], and remarkably, is also critical for subduing experimental colitis and autoimmune destructive brain changes in experimental allergic encephalitis [33], [34]. The data here supports dual agent receptor and protective functions for PrP. Although PrP appears to be the essential host molecule for agent binding and cell-to-cell spread, PrP, as well as its PrP-res form, can also be crucial for arresting agent production [23], and for eliminating infectious particles as demonstrated here. The binding of infectious TSE agent particles to PrP is a double-edged sword.

The very prolonged 200 day culture studies further support a role for PrP in innate defense mechanisms. Most notably, the continued production of elevated levels of both PrP and PrP-res ultimately led to the elimination of 4 logs of infectivity, with even fewer infectious particles than in baseline proliferative SEP cells. A loss of titer was expected in the cells returned to proliferative conditions. However, the continued production of clearly abnormal 5x levels of PrP was completely unexpected because1x baseline PrP was immediately restored in chronically arrested uninfected controls [22]. Even more surprising was the continued enormous production of PrP-res molecules (40% of the total PrP) in these “cured” cells. PrP-res continued to “be converted” and to accumulate even though PrP-res should have been diluted out a million fold during the 10 passages prior to assay. The intracellular mechanisms underlying this continued accumulation are not known, but could involve propagated seeded misfolding. While one cannot exclude additional SEP cell responses that ultimately led to the elimination of infectious particles between 140–200 days, PrP-res conversion appears to be involved in this process, especially because it continued unabated and disproportionately with respect to the negligible residue of infectious particles.

A dramatic agent strain change, rather than one or more host cell changes, is unlikely to account for the relatively rapid 60 day loss of infectious particles, especially when compared to the high agent accumulation during the first 140 days. Firstly, by 140 days there was very high multiplicity of infection, and homogeneous changes to the agent would have to affect virtually all of these particles in many cells. Notably, high dilution agent cloning in multiple species is required to achieve any substantial permanent strain change [reviewed in 2,21,23], and no cell culture strain change has shown either reproducible or comparable stable agent changes in serial animal passages, species susceptibility, and regional neuropathology. Second, there was no evidence of a change in PrP and PrP-res banding patterns that would denote a new variation in agent binding to PrP or strain. Indeed, the FU-CJD agent shows a geographic and strain specific characteristic 13kd band in GT1 cells that distinguishes it from non-Asiatic CJD strains and sheep scrapie strains [3], [9]. This same band was present in all the infected samples, including 200 day SEP cells with negligible infectivity. Third, the FU-CJD agent has been passaged over 30 times in mice and has exhibited no change in its ability to infect murine cells here after rat passages; a change after only 60 days in culture would, moreover, contrast with the lack of agent change by culture for much longer periods of >1 year [9]. Finally, even the highly cloned and passaged 263 K agent, selected for its inability to re-infect mice, does not completely lose its ability to permanently infect mice, murine cells and even Tga20 high murine PrP mice [3].

While the reasons for agent elimination by SEP cells are not known, and are of particular interest in the context of innovative therapeutic approaches to TSEs, the continued high production of both PrP and PrP-res under chronic arresting conditions implicates a self-perpetuating and escaped innate immune mechanism. Arrested cells at 200 days, with even higher PrP and PrP-res than their 140 day precursors, should have continued to produce high levels of infectivity. But these selected dividing cells also eliminated virtually all of their infectious particles, again suggesting that PrP-res is part of a host defense response that can become inappropriate, excessive and unstoppable. While PrP-res, a truncated amyloid form of the host protein, can trap infectious particles in its aggregate matrix, and limit their release, the continued production of enormous levels of PrP-res after the FU-CJD agent is cleared has major ramifications for other neurodegenerative diseases, including Alzheimer’s Disease (AD). In AD the initiating cause of amyloid formation remains largely unknown. AD pathology is likely to be initiated by various types of cumulative insults, including toxins, physical stresses, and infectious environmental agents. Alzheimer’s brain samples have never shown reproducible transmissibility, unlike CJD [26]. However, that does not rule out an infectious origin for at least a subset of AD cases. The above data show that even after an infection is cured, it can leave behind an active and perpetuating innate immune response that is inappropriate. Indeed, we previously noted that past viral infections, as in von Economo’s encephalitis (the “1918” influenza pandemic) represent “hit and run” viruses that apparently disappear clinically, but nevertheless can initiate or prime potential protein aggregation and misfolding diseases in older age; in addition, apparently innocuous viral sequences present in human brains (as the JC papova virus and paramyxoviruses) may also trigger or prime abnormal protein aggregates [27]. The original realization of post-encephalitic Parkinson’s disease was based on typical viral induced glial nodules in the substantia nigra of brains of people dying acutely during the pandemic, with survivors developing post-encephalitic tau positive neurofibrillary tangles (NFT) that are also present in AD, in traumatic dementia pugilistica, and in subacute persistent measles infections (subacute sclerosing pan-encephalitis). Post-encephalitic changes, as classic pre-senile AD and TSEs, also entail profound premature neuronal dropout. We propose that past and “cured” viral infections, some of which may appear to be innocuous, can also lead to a progressive cascade of amyloid changes in membrane proteins such as PrP and APP (the precursor of AD amyloid) that originate as part of an innate protective response against environmental pathogens. In terms of public health, a greater concentration on environmental insults could lead to more effective preventive initiatives in a broad population.
